# Association between Leisure Screen Time and Junk Food Intake in a Nationwide Representative Sample of Spanish Children (1–14 Years): A Cross-Sectional Study

**DOI:** 10.3390/healthcare9020228

**Published:** 2021-02-18

**Authors:** Àurea Cartanyà-Hueso, Adrián González-Marrón, Cristina Lidón-Moyano, Esteve Garcia-Palomo, Juan Carlos Martín-Sánchez, Jose M. Martínez-Sánchez

**Affiliations:** Group of Evaluation of Health Determinants and Health Policies, Department of Basic Sciences, Universitat Internacional de Catalunya, 08159 Sant Cugat del Vallès, Spain; acartanya@uic.es (À.C.-H.); clidon@uic.es (C.L.-M.); estevegarcia@uic.es (E.G.-P.); jcmartin@uic.es (J.C.M.-S.); jmmartinez@uic.es (J.M.M.-S.)

**Keywords:** child, child, preschool, diet, western, screen time, Spain

## Abstract

Evidence on the association between new patterns of leisure screen time and junk food consumption in Spanish children at the national level is scarce. The aim of this study is to assess the relation between daily leisure screen time and the frequency of sweet, soft drink, fast food, and snack intake in a representative sample of Spanish children and adolescents aged from 1 to 14 years. We conducted a cross-sectional study using a representative sample of the Spanish population under 15 years recruited for the 2017 Spanish National Health Survey (n = 5480). We dichotomized sweet, soft drink, fast food, and snack intake (high/low) and categorized daily leisure screen time (0–59, 60–119, 120–179, and ≥180 min). We calculated crude prevalence ratios and adjusted prevalence ratios, and their 95% confidence intervals (95% CI), of high frequency of sweet, soft drink, fast food, and snack intake. Children spending at least one hour of daily leisure screen time had higher prevalence of high frequency of sweet and snack intake than children being exposed less than one hour. For soft drinks and fast food, prevalence of high frequency intake was significantly higher from two and three hours of exposure, respectively. Longer periods of screen exposure in Spanish children during their leisure time may be associated with poorer dietary behaviors. The negative effects of excessive screen time in pediatrics population should be further studied.

## 1. Introduction

Childhood overweight and obesity are two of the current main public health issues worldwide. Around 38 million children aged under 5 and over 340 million children and adolescents aged 5–19 years were overweight or obese worldwide in 2019 and 2016, respectively [1]. In Europe, between the years 2016 and 2017, figures were just as alarming, with prevalences of overweight and obesity ranging from 10% to 21% in children aged 6 to 9 years, finding the highest prevalences in Southern European countries [2]. This is in line with the fact that, in Spain, between the years 2014 and 2015, around 30% of children between 3 and 18 were overweight or obese [3].

Importantly, children who are obese are more likely to have health issues over their childhood and later in their adulthood [4,5,6,7,8,9]. Childhood overweight and obesity are associated with a combination of some genetic, behavioral, and environmental factors [9], which cause an energy imbalance between calories consumed and calories expended [1]. A systematic review published in 2020 shows that unhealthy lifestyle behaviors, including higher patterns of screen time, unhealthy dietary habits, and lower levels of physical activity during childhood are associated with adiposity risk [10]. In this sense, dietary patterns have become of poorer quality over the last years mainly due to a reduction in the adherence to the Mediterranean Diet, which evidence shows is a protective factor for adiposity [11], and an increase in the intake of junk food, defined as food that is unhealthy but is easy to eat [12], which might be due to Western influences [13,14,15]. Although childhood overweight and obesity are currently a pandemic, the consequences of junk food consumption go beyond. Junk food consumption at an early age has been associated with other physical conditions, such as metabolic syndrome [16], and also with behavioral problems, including hyperactivity [17], psychiatric distress, or violence [18].

Different systematic reviews [19,20] show a positive relationship between screen time, based on TV viewing, and unhealthier dietary patterns. Further, there is increasing evidence that screen time is related to a great variety of physiological and psychological issues [21]. A recent systematic review of reviews on the association between screen time and health outcomes in children and adolescents concluded that there is strong evidence on the association between screen time and adiposity, unhealthy diet, depressive symptoms, and quality of life, while evidence for other outcomes (e.g., cardiovascular risk and fitness) is weak [21]. Data on other forms of screen time different from television (e.g., computer, video, and mobile phones) were very sparse in these systematic reviews, reflecting the lack of evidence on the effects that new screen time patterns have on children’s health. Currently there is, however, a constant increase in screen time exposure in children, which has prompted a number of worldwide and national health institutions to provide guidelines following the same definition of proper daily screen time: to avoid screen exposure for children under 2 years, to limit screen time to 1 h for children between 2 and 4 years, and to limit leisure screen time (i.e., screen time spent on non-educational issues) up to 2 h for children and adolescents between 5 and 17 years [22,23,24].

As mentioned above, the great amount of evidence regarding the association between health outcomes and lifestyle choices, including dietary patterns, and leisure screen time in children is not representative of the current screen time usage. In Spain concretely, this limitation is also found in the “Food, Physical Activity, Child development and Obesity” study (in Spanish: “Alimentación, Actividad Física, Desarrollo Infantil y Obesidad”) ALADINO study [25], the largest study carried out, as far as we know, at the national level assessing the association between screen time and food and drink consumption, which took into account the exposure to TV, computers, and video games only, leaving out tablets and smartphones, since this study was carried out in 2011 and 2013. For the last five years, however, the prevalence of owning a mobile phone in Spain in children aged from 10 to 15 is higher than 65% [26,27,28,29,30].

Therefore, the aim of this study is to assess the relation between daily leisure screen time and the frequency of eating sweets, soft drinks, fast food, and snacks in a representative sample of Spanish children and adolescents aged from 1 to 14 years.

## 2. Methods

This is a cross-sectional study using a representative sample of the Spanish population under 15 years recruited for the 2017 Spanish National Health Survey (2017SNHS) (n = 6106). Data were collected between October 2016 and October 2017. Participants were selected through a stratified three-stage sampling, where the first stage was the census tracts and the second stage was the main family dwellings. Within each household, a participant aged 15 or over was selected to complete the Adult Questionnaire. If a children (aged 0–14) also lived in the household, a Child Questionnaire was also completed. The third stage was the individuals. The response rate for this survey was 72.2%. All the information was obtained through a computer-assisted personal interviewing answered by any of the parents, the legal guardian or other relative in the case of impossibility of the former [31]. Exclusion criteria for our study were (1) being under 1 year, as questions of screen time and junk food intake were not applicable for these children (n = 256); (2) having a limitation for doing any activity usually carried out by children due to any health issue, as reported by the person who answered the questionnaire (n = 332); and (3) being diagnosed by a doctor with diabetes, as diabetic children should control the sugar intake (n = 12), and having missing data in all outcome variables and/or the exposure variable (n = 26). Thus, the final sample included 5480 Spanish children from 1 to 14 years.

### 2.1. Research Ethics

The investigation was carried out considering the rules of the Declaration of Helsinki of 1975. Written informed consent was not necessary since anonymous data were used. This manuscript is part of a PhD thesis approved by the Ethics Committee of the Universitat Internacional de Catalunya with code CBAS-2020-06.

### 2.2. Frequency of Sweet, Soft Drink, Fast Food, and Snack Intake

Data on the frequency of sweet, soft drink, fast food, and snack intake were retrieved through the question “How often does the child eat each of the following groups of food?”, with six possible responses (once or more than once a day, from 4 to 6 times a week, 3 times a week, once or twice a week, less than once a week, and never). We dichotomized the responses to the variables frequency of sweet, soft drink, fast food, and snack consumption as (a) high: respondents who answered that the child eats more than twice a week, and (b) low: otherwise.

### 2.3. Daily Leisure Screen Time

The questionnaire collected the leisure screen time through the question “Approximately, during the leisure time of the child in a day, how much time does the child spend in front of the screen, including computer, tablet, TV, videos, videogames, or cell phone?” differentiating between two periods of time: from Monday to Friday and from Saturday to Sunday (an independent question for each of the two periods). Respondents answered among three possible responses (never or almost never, less than 1 h, and at least 1 h). Then, those respondents that answered at least 1 h in any period of time, also reported the daily number of hours of leisure screen time, ranging from 1 h to 12 h, in the corresponding period of time. Next, we calculated the daily number of hours of leisure screen time through a weighted mean. We categorized the daily leisure screen time in minutes in 4 categories (0–59, 60–119, 120–179, and ≥180). Finally, we calculated the daily leisure screen time in hours after one hour of exposure.

### 2.4. Potential Confounding Variables and Covariates

Based on literature [10,20,32,33,34,35,36,37], we used the following variables as potential confounders: (1) sex (male and female) and (2) age of the child in years (1–2, 3–5, 6–11, and 12–14), categorized according to the Spanish school system, as we assume that children in different school stages have different routines, screen time usages, and dietary patterns; (3) education level of the parents (low, medium, and high); (4) relationship between who answered the questionnaire and the child (parents and others); (5) family structure (couple-parent, single-parent, couple-parent or single-parent living with other family members, and others); (6) sleep duration (proper sleep duration and short sleep duration), dichotomized according to the recommendations of the National Sleep Foundation (NSF) [38,39]; (7) physical activity in their leisure time (no exercise, physical or sports activity occasionally, physical activity several times a month, sports or physical training several times a week); (8) Body Mass Index for age (BMI-for-age) z-score, which is the standardized measure through weight and height reported by person who answered the questionnaire, calculated according to the child growth standards of the World Health Organization (WHO) [40]. We treated as missing data those z-scores classified as Biologically Implausible value (BIV) [41,42,43] for weight-for-age, height-for-age, and BMI-for-age; and (9) proxy of parent frequency of sweet, soft drink, fast food, and snack intake (high and low). Last, data were obtained through the answers of a person over 14 years that lived together with the child and answered the adult questionnaire. Due to the methodology of the 2017SNHS, we could not identify if that person was any of the parents, but around 93% of children’s families consisted in a couple or single-parent family. Therefore, we assume that most of persons responding the adult questionnaire was a parent.

### 2.5. Statistical Analysis

We described the outcomes, the exposure, potential confounding variables, and covariates. We computed absolute and relative frequency for categorical variables and mean and standard deviation or median and interquartile range for numeric variables. We calculated the crude prevalence ratios (PR) and adjusted prevalence ratios (aPR), and their 95% confidence intervals (95% CI), of high frequency of sweet, soft drink, fast food, and snack intake according to the daily leisure screen time, taking as reference the category 0–59 min, and according to the daily leisure screen time after one hour of exposure. We also calculated the PR and aPR, and their 95% CI, of high frequency of soft drink, fast food, and snack intake, combined two by two, and the combination of the three together, according to the daily leisure screen time, taking as reference the category 0–59 min, and according to the daily leisure screen time after one hour of exposure. Models were fitted by generalized linear models with Poisson family and robust variances [44]. Associations were adjusted for potential confounding variables. Statistical software used was R-3.5.2.

## 3. Results

The median age of the children was 8 years (interquartile range: 4–11), 51.9% were male, 78.1% lived with a couple as family structure, 76.5% had proper sleep duration, and 21.6% did no exercise. An estimated 29.3%, 26.4%, 28.6%, and 15.7% of children spent less than 60, from 60 to 119, from 120 to 179, and at least 180 min of daily leisure screen time, respectively (Table 1). In addition, an estimated 29.0% of children between 12 and 14 years had at least 180 min of leisure screen time daily. Percentages decrease to 14.3%, 9.1%, and 4.9% for children between 6 and 11, 3–5, and 1–2 years, respectively. The average fruit and vegetable intake was 1.2 times and 0.66 times per day, respectively. An estimated 93.9% and 88.4% of children in our sample take dairy products and bread or cereals more than once daily. The highest percentage of high frequency of sweet intake, the group of junk food with a highest prevalence of consumption, corresponds to children between 3–5 years, with an estimated 78.1%.

### 3.1. Association between Daily Leisure Screen Time and Junk Food Intake

The largest prevalence of high frequency of consumption among the four outcomes analyzed corresponds to sweet intake, with 75% of children. Table 2 shows that the adjusted prevalence of high frequency of sweet and snack intake for children having at least one hour of daily leisure screen time are significantly higher than those for children having less than an hour of daily leisure screen time. The adjusted prevalence of high frequency of soft drink intake is significantly higher for children having at least two hours of exposure than that for children having less than an hour of exposure. For fast food, the adjusted prevalence from three hours of exposure is significantly higher than that for children having less than an hour of daily leisure screen time. Increasing point estimates for the prevalence of high risk in the four outcomes are generally observed for longer periods of daily leisure screen time.

Table 3 shows that the adjusted prevalence of high frequency of composite intakes for children having at least three hours of daily leisure screen time range from 2.29 (CI 95% 1.44,3.65) to 4.27 (CI 95% 2.03,8.96) times that those for children having less than an hour of daily leisure screen time.

### 3.2. Association between Daily Leisure Screen Time after One Hour of Exposure and Junk Food Intake

The prevalence of high frequency of snack intake increased around 25% for every hour of daily leisure screen time after one hour of exposure. Significant increases in the prevalence of high frequency of sweet, fast food, and soft drink intake after one hour of exposure are also observed. For composite intakes, aPR range from 1.31 (95% CI 1.20,1.43) to 1.45 (95% CI 1.31,1.61) (Table 2 and Table 3).

## 4. Discussion

Children spending at least one hour of daily leisure screen time had higher prevalence of high frequency of sweet and snack intake than children being exposed less than one hour. For soft drinks and fast food, prevalence of high frequency intake was significantly higher from two and three hours of exposure, respectively. Further, the prevalence of high frequency of junk food consumption significantly increased for every hour of daily leisure screen time after the first hour of exposure.

Our results update, and add to the evidence, that longer periods of screen time may be associated with higher prevalences of junk food consumption. Mainly, our results are in line with those of the 2011 and 2013 ALADINO study [25], which included a representative sample of Spanish children from six to nine and from seven to eight years, respectively, and found that higher levels of screen time were associated with a larger frequency of consumption of energy-dense products. In other studies in which the association between Mediterranean Diet and screen exposure was assessed in Spanish children [15,45,46], longer periods of screen time were associated with a lower adherence to the Mediterranean Diet. This is consistent with our results, as the Mediterranean Diet is characterized by a low intake of junk food.

We have also found that children exposed to screens in their leisure time for at least 180 min daily have a higher frequency of different combinations of the variables of consumption. Remarkably, these associations were, in general, stronger than with the individual outcomes. This may indicate that screen exposure is associated not only with isolated unhealthy dietary behaviors but with broader, unhealthy nutritional patterns. Therefore, our results enforce the results obtained in previous systematic reviews [19,20].

Prevalence of childhood overweight and obesity are on the rise worldwide at present. In Spain, over 30% of children between three and eighteen are obese or overweight [3]. Among the plethora of social, biological, environmental, and economic determinants associated with these conditions, screen time exposure may play an important role not only because it may be associated with poorer dietary patterns, but also because it may act as a displacer of healthier habits, such as doing exercise. In this sense, and although it is not the main aim of the study, we have also found that children doing sports or physical training several times a week have lower adjusted prevalence of soft drink (aPR: 0.63, 95% CI: 0.52, 0.77) and fast food intake (aPR: 0.64, 95% CI: 0.47, 0.88) than children who do not do exercise. Physical activity has been proposed to be a stress-induced eating repressor, which may eventually limit junk food consumption [47]. Furthermore, greater screen time exposure has been found to be associated with lower levels of physical activity in Spanish children [48], meaning that physical activity could mediate in the association between screen time exposure and junk food consumption. The effect of increased levels of physical activity on the reduction of junk food consumption could also be explained by the displacement of screen time exposure, which results in a reduced junk food consumption (main association explored in this paper). In this case, screen time exposure could act as a mediator in the association between physical activity and junk food consumption.

### 4.1. Public Health Implications

One of the seventeen United Nations Sustainable Development Goals consists in ensuring good health and promoting well-being, with a special focus on children [49]. In this sense, further prospective studies should be carried out to confirm our results in order for governments to consider implementing comprehensive policies directed not only to reduce leisure screen time, but also to promote healthier habits overall which may impact children’s future health. Specific programs may include school-based interventions to encourage children to change their lifestyles or information campaigns for parents to inform them on the consequences of the potential harmful use of screen devices in their kids.

### 4.2. Strengths and Limitations

This is the first study assessing the relation between dietary patterns and screen time in a representative sample of Spanish pediatric population including in the overall screen time smartphone and tablet use, as well as considering combinations of different junk food categories. Our study contains some limitations that should be mentioned. First, this study does not differentiate between different types of screen exposure, which may have different effects on junk food consumption, and may hamper the potential implementation of focused public health interventions directed to reduce specific screen exposures. Additionally, due to the cross-sectional design of the survey, a causal effect of leisure screen time on the frequency of junk food consumption cannot be established and reverse causality should not be discarded. However, we hypothesize that screen time may be associated with junk food consumption in our study through different pathways. First, evidence shows that children belonging to low-income families are more likely to spend longer periods using screens and follow worse dietary patterns [10] as parents have longer working hours, less time to look after their children, and are less informed on the harmful effects of low-quality diets. In this sense, parents having longer working hours may offer their children more readily available options requiring less of their time, such as mobile devices to entertain them and junk food to feed them. Furthermore, children may be more exposed to junk food advertisements while on screen time, triggering its consumption. Also, screen time may be associated with increased levels of stress, which may turn into stress-induced eating and subsequent junk food consumption. Finally, inaccurate estimates of junk food consumption and daily leisure screen time may have been reported, leading to information bias. This may be more relevant for older children due to their more independent lives (i.e., respondents could be unaware of certain intakes) and associated with a response bias from parents which could be reduced in future investigations with the use of dietary records and real-time monitoring.

## 5. Conclusions

The results of the present investigation suggest that longer periods of screen exposure in Spanish children during their leisure time may be associated with poorer dietary behaviors. This highlights the necessity to continue studying the potential negative effects of excessive screen time in pediatrics population for Spanish health institutions to consider promoting interventions to control its use. These findings, however, should be confirmed in prospective studies.

## Figures and Tables

**Table 1:** Characteristics of the study sample.

Variables of the Study (n = 5480)	n (%)/Mean (SD)
**Sex of the child**	
Male	2845 (51.9)
Female	2635 (48.1)
**Age of the child (years)**	
1–2	729 (13.3)
3–5	1038 (18.9)
6–11	2349 (42.9)
12–14	1364 (24.9)
**Education level**	
Low	453 (8.6)
Medium	3031 (57.4)
High	1799 (34.0)
**Relationship between who answered** **the questionnarie and the child**	
Parents	308 (5.6)
Other	5172 (94.4)
**Family structure**	
Couple	4281 (78.1)
Parent-single family	603 (11.0)
Couple or parent-single family with other family members	436 (8.0)
Others	160 (2.9)
**Sleep duration**	
Proper sleep duration	4190 (76.5)
Short sleep duration	1290 (23.5)
**Physical activity**	
No exercise	1181 (21.6)
Physical or sports activity occasionally	1361 (24.9)
Physical activity several times a month	1414 (25.8)
Sports or physical training several times a week	1504 (27.5)
**BMI-for-age zscore**	0.51 (1.54)
**Frequency of sweets proxy of parents**	
Low	2479 (51.8)
High	2308 (48.2)
**Frequency of soft drinks proxy of parents**	
Low	1089 (22.7)
High	3699 (77.3)
**Frequency of fast food proxy of parents**	
Low	376 (7.9)
High	4409 (92.1)
**Frequency of snacks proxy of parents**	
Low	415 (8.7)
High	4370 (91.3)
**Daily leisure screen time (min)**	
0–59	1605 (29.3)
60–119	1444 (26.4)
120–179	1570 (28.6)
≥180	861 (15.7)
**Daily leisure screen time after** **one hour of exposure (hours)**	0.9 (1.1)
**Sweet intake**	
Low	1372 (25.0)
High	4106 (75.0)
**Soft drink intake**	
Low	4759 (87.0)
High	708 (13.0)
**Fast food intake**	
Low	4958 (90.6)
High	515 (9.4)
**Snack intake**	
Low	4869 (89.0)
High	604 (11.0)

n: absolute frequency; %: percentage; SD: standard deviation; BMI-for-age: Body Mass Index for age; min: minutes.

**Table 2:** Frequency of the categories of the variable daily leisure screen time and crude and adjusted prevalence ratios for the variables high frequency of sweet, soft drink, fast food, and snack intake and for daily leisure screen time after one hour of exposure according to the daily leisure screen time.

	High Frequency of Sweets (n = 5478)			High Frequency of Soft Drinks (n = 5467)			High Frequency of Fast Food (n = 5473)			High Frequency of Snacks (n = 5473)		
	**%**	**PR (95% CI)**	**aPR (95% CI)**	**%**	**PR (95% CI)**	**aPR (95% CI)**	**%**	**PR (95% CI)**	**aPR (95% CI)**	**%**	**PR (95% CI)**	**aPR (95% CI)**
**Daily leisure screen** **time (min)**												
0–59	69.2	1.00 Reference	1.00 Reference	7.9	1.00 Reference	1.00 Reference	8.1	1.00 Reference	1.00 Reference	7.9	1.00 Reference	1.00 Reference
60–119	76.1	1.10 (1.05,1.15)	1.05 (1.01,1.10)	9.5	1.21 (0.96,1.52)	1.09 (0.86,1.39)	7.6	0.93 (0.73,1.19)	0.83 (0.65,1.06)	9.0	1.15 (0.91,1.45)	1.30(1.00,1.68)
120–179	77.1	1.11 (1.07,1.16)	1.05 (1.01,1.10)	14.1	1.79 (1.45,2.20)	1.31 (1.05,1.63)	8.7	1.07 (0.85,1.35)	0.84 (0.67,1.07)	11.0	1.39 (1.12,1.74)	1.35 (1.06,1.73)
≥ 180	79.9	1.15 (1.10,1.21)	1.09 (1.03,1.14)	26.1	3.31 (2.71,4.05)	1.83 (1.47,2.27)	16.2	1.99 (1.59,2.49)	1.38 (1.08,1.75)	20.5	2.60 (2.10,3.22)	2.36 (1.84,3.02)
	**Mean (SD)**	**PR (95% CI)**	**aPR (95% CI)**	**Mean (SD)**	**PR (95% CI)**	**aPR (95% CI)**	**Mean (SD)**	**PR (95% CI)**	**aPR (95% CI)**	**Mean (SD)**	**PR (95% CI)**	**aPR (95% CI)**
**Daily leisure screen time** **after one hour of exposure** **(hours)**	0.9 (1.1)	1.04 (1.03,1.05)	1.02 (1.01,1.04)	1.4 (1.3)	1.40 (1.34,1.46)	1.20 (1.15,1.26)	1.3 (1.4)	1.28 (1.21,1.36)	1.15 (1.08,1.22)	1.3 (1.3)	1.31 (1.25,1.38)	1.25 (1.19,1.31)

%: percentage; PR: crude Prevalence Ratio; aPR: adjusted Prevalence Ratio; 95% CI: 95% Confidence Interval; SD: Standard Deviation; min: minutes.

**Table 3:** Frequency of the categories of the variable daily leisure screen time and crude and adjusted prevalence ratios for the composite variables of high frequency of sweet, soft drink, fast food, and snack intake and for daily leisure screen time after one hour of exposure according to the daily leisure screen time.

	High Frequency of Soft Drinks and Fast food (n = 5466)			High Frequency of Soft Drinks and Snacks (n = 5466)			High Frequency of Fast Food and Snacks (n = 5470)			High Frequency of Soft Drinks, Fast Food, and Snacks (n = 5465)		
	**%**	**PR (95% CI)**	**aPR (95% CI)**	**%**	**PR (95% CI)**	**aPR (95% CI)**	**%**	**PR (95% CI)**	**aPR (95% CI)**	**%**	**PR (95% CI)**	**aPR (95% CI)**
**Daily leisure screen** **time (min)**												
0–59	1.5	1.00 Reference	1.00 Reference	1.8	1.00 Reference	1.00 Reference	1.8	1.00 Reference	1.00 Reference	0.6	1.00 Reference	1.00 Reference
60–119	2.0	1.34 (0.78,2.29)	1.43 (0.82,2.48)	2.6	1.51 (0.93,2.44)	1.51 (0.91,2.50)	1.9	1.11 (0.66,1.87)	0.90 (0.53,1.53)	1.2	1.89 (0.87,4.11)	1.83 (0.84,3.96)
120–179	2.4	1.61 (0.97,2.67)	1.22 (0.71,2.09)	3.5	2.00 (1.28,3.14)	1.48 (0.92,2.39)	2.6	1.49 (0.93,2.40)	0.97 (0.58,1.60)	1.3	2.14 (1.01,4.52)	1.42 (0.65,3.13)
≥ 180	7.9	5.28 (3.34,8.34)	3.20 (1.91,5.33)	9.6	5.46 (3.58,8.31)	3.11 (1.95,4.94)	7.4	4.25 (2.75,6.58)	2.29 (1.44,3.65)	5.0	8.01 (4.04,15.86)	4.27 (2.03,8.96)
	**Mean (SD)**	**PR (95% CI)**	**aPR (95% CI)**	**Mean (SD)**	**PR (95% CI)**	**aPR (95% CI)**	**Mean (SD)**	**PR (95% CI)**	**aPR (95% CI)**	**Mean (SD)**	**PR (95% CI)**	**aPR (95% CI)**
**Daily leisure screen time** **after one hour of exposure** **(hours)**	1.8 (1.6)	1.59 (1.46,1.73)	1.42 (1.30,1.54)	1.6 (1.4)	1.52 (1.41,1.64)	1.32 (1.23,1.42)	1.6 (1.5)	1.51 (1.39,1.65)	1.31 (1.20,1.43)	1.9 (1.5)	1.65 (1.48,1.82)	1.45 (1.31,1.61)

%: percentage; PR: crude Prevalence Ratio; aPR: adjusted Prevalence Ratio; 95% CI: 95% Confidence Interval; SD: Standard Deviation; min: minutes.
